# UnERFing auxin-mediated degradation in the emerging lateral root

**DOI:** 10.1093/plcell/koae079

**Published:** 2024-03-12

**Authors:** Rory Osborne

**Affiliations:** Assistant Features Editor, The Plant Cell, American Society of Plant Biologists, USA; School of Biosciences, University of Birmingham, Birmingham B15 2TT, UK

The development of lateral roots (LRs) was essential for the successful colonization of land by vascular plants. These roots play an important role in acquiring water and nutrients, facilitating below-ground exploration, and aiding in anchoring both young and established plants to their growth substrate. LR formation is so important, in fact, that it is a defining feature of all extant euphyllophytes (Hetherington et al. 2020). Understanding root plasticity and the regulatory mechanisms controlling the seemingly spontaneous formation of these post-embryonic organs therefore goes hand-in-hand with our understanding of plant adaptation and evolution.

As the running joke goes, the phytohormone auxin is at the root of all plant growth and development, and LR formation is no exception. Auxin plays a critical role in LR patterning, their initiation in xylem pole pericycle cells, and the formation of lateral root primordia, while the AUXIN RESPONSE FACTORs ARF7 and ARF19 are responsible for LR initiation. However, there are still many unanswered questions about how this complicated process is regulated, and unknown players are yet to be revealed. In this issue of *The Plant Cell*, **Zipeng Yu, Xingzhen Qu, and colleagues (Yu et al. 2024)** beautifully characterize the mechanism underpinning the auxin-dependent post-translational regulation of ETHYLENE RESPONSE FACTOR 13 (ERF13) (see Fig. 1). The authors previously showed that ERF13 is degraded in an auxin-dependent manner and negatively regulates LR formation (Lv et al. 2021). In this study, they explore how this degradation is regulated. In doing so, they reveal a novel role for MOS4-ASSOCIATED COMPLEX 3A (MAC3A) and 3B (MAC3B), so far only characterized as regulators of the plant immune response (Monaghan et al. 2009), in promoting the emergence of LRs.

**Figure 1.**
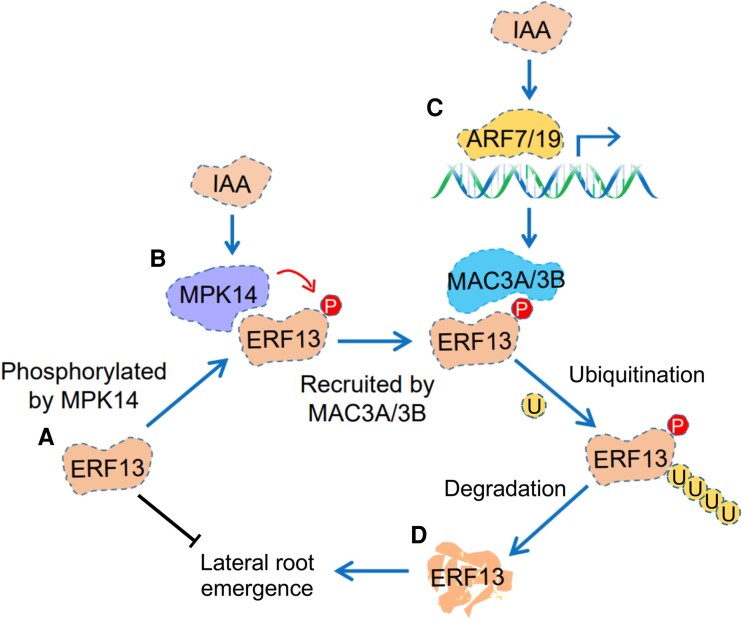
Proposed model of auxin-mediated regulation of ERF13 degradation in the emerging lateral root. **A)** ERF13 represses lateral root emergence. **B)** IAA activated MPK14 phosphorylates (P) ERF13 promoting its interaction with MAC3A/3B. **C)** IAA activates ARF7/19, inducing expression of *MAC3A/3B*, which targets phosphorylated ERF13 for ubiquitination (U). **D)** Ubiquitinated ERF13 is degraded, releasing its repressive effect on lateral root emergence. Adapted from Yu et al. (2024), Figure 8.

The authors' first aim was to identify interactors of ERF13 that might explain its auxin-dependent degradation. Performing immunoprecipitation-mass spectrometry on *35S::ERF13-MYC-*expressing *Arabidopsis thaliana* seedlings, they observed the enrichment of 2 E3 ligases, MAC3A and its homologue MAC3B. This interaction was confirmed in planta and was remarkably stronger when the U-box domain of MAC3A/B had been deleted, suggesting these E3s might target ERF13 for degradation. Microscopy and histochemical staining of tagged *MAC3A/B*-expressing plants revealed clear enrichment of these proteins in emerging lateral roots (particularly stage IV), while a *mac3a/b* double mutant produced significantly fewer LRs vs control plants. These results prompted the authors to investigate whether these 2 E3 ligases regulate ERF13 post-translationally to coordinate LR development.

Using several well-controlled co-immunoprecipitations, MAC3A and MAC3B were shown to act redundantly in ubiquitinating ERF13, which was then degraded by the proteasome. Most interestingly, however, was that the interaction between these proteins was enhanced under auxin treatment. In their previous work, the authors showed that ERF13 was phosphorylated under high levels of auxin, dependent on MAP-KINASE 14 (MAPK14) (Lv et al. 2021). Using phospho-mimic (ERF13^DDD^) and non-phosphorylatable (ERF13^AAA^) variants of ERF13, Yu et al. demonstrate that MAC3A/B possess weaker affinities to ERF13^AAA^ and require ERF13 phosphorylation to ubiquitylate it in the first place.

To complete the regulatory picture, the authors investigated whether auxin directly regulates *MAC3A* or *MAC3B*. While auxin treatment induced the expression of both genes and promoted the accumulation of both proteins at LR primordia, this effect was attenuated in *arf7* and *arf19* mutants, suggesting these E3 ligases are downstream targets of these master regulators of LR initiation. Thus, the authors conclude that auxin regulates ERF13 via 2 mechanisms: first, it activates MAPK14, which then phosphorylates ERF13, increasing its binding affinity to MAC3A/B, and secondly, it promotes expression of *MAC3A/B* at sites of LR initiation. Together this results in the rapid and precise degradation of ERF13, releasing its repressive role to facilitate LR formation. Considering the *mac3a/b* double mutant was reported to be smaller than control plants (Li et al. 2018), it is also possible that ERF13, or other unidentified targets of MAC3A/B, negatively regulate growth in foliar tissues. Therefore, having identified another piece to the puzzle of the complex regulatory network controlling lateral root formation, it will be important to identify other ubiquitination targets of MAC3A or MAC3B and determine whether these also contribute to LR development.

## Dive Curated Terms

The following phenotypic, genotypic, and functional terms are of significance to the work described in this paper:

ARF19 Gramene: AT1G19220
ARF19 Araport: AT1G19220

ARF7 Gramene: AT5G20730
ARF7 Araport: AT5G20730

DDD CHEBI: CHEBI:27841

MAC3B Gramene: AT2G33340
MAC3B Araport: AT2G33340

ERF13 Gramene: Mp6g08690.1
ERF13 Araport: Mp6g08690.1

MAC3A Gramene: AT1G04510
MAC3A Araport: AT1G04510
